# 25-Hydroxy- and 1α,25-Dihydroxycholecalciferol Have Greater Potencies than 25-Hydroxy- and 1α,25-Dihydroxyergocalciferol in Modulating Cultured Human and Mouse Osteoblast Activities

**DOI:** 10.1371/journal.pone.0165462

**Published:** 2016-11-28

**Authors:** Allahdad Zarei, Philippa A. Hulley, Afsie Sabokbar, M. Kassim Javaid, Alireza Morovat

**Affiliations:** 1 Botnar Research Centre, Nuffield Department of Orthopaedics, Rheumatology and Musculoskeletal Sciences, University of Oxford, Oxford, United Kingdom; 2 Department of Clinical Biochemistry, Oxford University Hospitals, Oxford, United Kingdom; Universite de Lyon, FRANCE

## Abstract

Despite differences in the phamacokinetics of 25-hydroxycholecalciferol (25(OH)D_3_) and 25-hydroxyergocalciferol (25(OH)D_2_) in man, the effects of these and their 1α-hydroxylated forms (1,25(OH)_2_D_3_ and 1,25(OH)_2_D_2_) on cellular activity of vitamin D-responsive cells have hardly been compared. We studied differences in the effects of these metabolites on cell number, gene transcription, protein expression and mineralisation of cultured human bone marrow-derived stromal cells (hBMSC) and rapidly mineralising mouse 2T3 osteoblasts. 50–1000 nM 25(OH) and 0.05–10 nM 1,25(OH)_2_ metabolites were used. At high concentrations, 25(OH)D_2_/D_3_ and 1,25(OH)_2_D_2_/D_3_ suppressed cell number in both human and mouse cells. The suppression was greater with cholecalciferol (D_3_) metabolites than with those of ergocalciferol (D_2_). In both cell types, 25(OH)D_2_ and 25(OH)D_3_ increased the expression of osteopontin, osteocalcin, collagen-1, receptor activator of nuclear factor kappa-B ligand, vitamin D receptor, CYP24A1 and CYP27B1 genes. Whereas there was little or no difference between the effects of 25(OH)D_2_ and 25(OH)D_3_ in hBMSCs, differences were observed in the magnitude of the effects of these metabolites on the expression of most studied genes in 2T3 cells. Alkaline phosphatase (ALP) activity was increased by 25(OH)D_2_/D_3_ and 1,25(OH)_2_D_2_/D_3_ in hBMSC and 2T3 cells, and the increase was greater with the D_3_ metabolites at high concentrations. In hBMSCs, mineralisation was also increased by 25(OH)D_2_/D_3_ and 1,25(OH)_2_D_2_/D_3_ at high concentrations, with D_3_ metabolites exerting a greater influence. In 2T3 cells, the effects of these compounds on mineralisation were stimulatory at low concentrations and inhibitory when high concentrations were used. The suppression at high concentrations was greater with the D_3_ metabolites. These findings suggest that there are differences in the effects of 25-hydroxy and 1α,25(OH)_2_ metabolites of D_3_ and D_2_ on human preosteoblasts and mouse osteoblasts, with the D_3_ metabolites being more potent in suppressing cell number, increasing ALP activity and influencing mineralisation.

## Introduction

Vitamin D (vit D) regulates bone function and its deficiency is associated with bone loss [1,2]. Vit D receptors (VDR) are present in almost all human cells, and the list of roles and functions of vit D has been expanding [3]. Osteoblasts, too, express VDR, although exactly how 1α,25-dihydroxycholecalciferol (1,25(OH)_2_D_3_) and 1α,25-dihydroxyergocalciferol (1,25(OH)_2_D_2_) act on these cells to regulate bone metabolism is not fully understood [4]. Osteoblasts differentiate from bone marrow-derived stromal cells (BMSCs) through several well-defined stages that include proliferation, maturation and mineralisation [5]. During the final extracellular matrix formation step, some osteoblasts get embedded in osteoid as osteocytes [6]. 1,25(OH)_2_D_3_ promotes differentiation of human BMSCs into osteoblasts, inhibits cell proliferation, and stimulates mineralisation in both human and mouse osteoblast cells [7–10]. Some of these effects are mediated through LRP5 and the Wnt signalling pathway [11,12], and the stimulation of mineralisation is partly secondary to an increase in osteoblastic alkaline phosphatase (ALP) activity [13]. The 1α,25-hydroxylated metabolites induce the expression of osteoblast signature genes, including collagen type-I (*col1a*), osteocalcin (*ocn*), osteopontin (*opn*), osteoprotegerin (*opg*), receptor activator of nuclear factor kappa-B ligand (*rankl*), *vdr* [10,14]. Thus, formation of COL1a and non-collagenous proteins, such as OCN and OPN, are stimulated by 1,25(OH)_2_D_3_, and osteoclastogenesis is inhibited by an increase in OPG, which binds RANKL and prevents its interaction with RANK on osteoclast precursors [8]. Osteoblasts also possess CYP27B1 enzyme, which 1α-hydroxylates 25-hydoxycholecalcifeol (25(OH)D_3_) and 25-hydoxyergocalcifeol (25(OH)D_2_) into 1,25(OH)_2_D_3_ and 1,25(OH)_2_D_3_, as well as 1α,25-dihydroxycholecalciferol 24-hydroxylase, CYP24A1. Osteoblasts are therefore capable of responding locally to 25-(OH)D and produce metabolites that act in an autocrine fashion [14,15].

Patients with vit D deficiency carry a risk of reduced bone mineral density and developing osteoporosis. With the recognition that large portions of populations living at high latitudes are deficient in vit D [16–18], a need to identify and treat the deficiency has increasing been realised. For the treatment, it has been generally assumed that the two prescribed forms of vit D, cholecalciferol (D_3_) and ergocalciferol (D_2_), have equal potencies. However, this has been questioned [19,20]. A few studies in humans have shown that lower circulating concentrations of 25(OH)D are achieved as a result of the administration of D_2_ compared with D_3_ [21,22]. Other studies have shown a lower binding to vit D-binding protein and a higher clearance rate for D_2_ metabolites compared with those of D_3_ [23,24], and differences in the effects of active D_2_ and D_3_ metabolites on plasma calcium and bone mineral content have been observed [25,26]. However, very few studies have compared potencies of 25(OH) and 1,25(OH)_2_ metabolites of D_2_ and D_3_ at the cellular level. Although 1,25(OH)_2_D_2_ and 1,25(OH)_2_D_3_ were shown in separate studies to have equal affinities for VDR in chick intestine, rat intestine, porcine kidney, human breast cancer cells and HeLa cells [27–29], 1,25(OH)_2_D_3_ was able to up-regulate rat intestinal VDR more than 1,25(OH)_2_D_2_ did [30], and differences in the effects of 1α-cholecalciferol and 1α-ergocalciferol were shown in rat intestinal calcium absorption, and osteoclast numbers and activity [31,32]. As for osteoblasts, to the best of our knowledge, no study has compared their response to 25(OH) or 1,25(OH)_2_ metabolites of D_2_ and D_3_.

Therefore, we have undertaken a comparison of the effects and potencies of 25(OH) and 1,25(OH)_2_ metabolites of D_2_ and D_3_ on osteoblast cell number, cellular activity and mineralisation. For this, we have used two very distinct cell types: primary human bone marrow-derived stromal cells (hBMSC) and mouse 2T3 osteoblastic cell line. Human BMSCs are at the initial stage of differentiation, and require osteogenic factors and time to develop into pre- and full osteoblasts. Based on our unpublished data, these cells require a period of 4 weeks to become responsive and to begin to mineralise, whereas murine 2T3 cells are primary murine osteoblasts that have been immortalised using SV40 large T antigen on the BMP2 promoter and that respond rapidly to matrix maturation and mineralisation signals.

## Material and Methods

### Cell culture

Human BMSCs were derived from discarded bone material from patients undergoing total hip replacement. The use of bone material was approved by the University of Oxford Musculoskeletal Biobank Ethical Committee in compliance with Human Tissue Act ethical guidelines, and was after obtaining written, informed consent from patients [33]. Human BMSCs were cultured and studied at passage 1–2 in α-Modified Eagle’s Minimum Essential Medium (αMEM, Lonza), supplemented with 10% heat-inactivated foetal bovine serum (HI-FBS; Lonza, UK), L-glutamine (2mM), ribonucleosides, penicillin and streptomycin (100U/ml).

Murine 2T3 pre-osteoblast cells were kind donations from Professor Stephen E. Harris (University of Texas, San Antonio, USA) and have been characterized previously [34]. These cells were isolated and cloned from transgenic mice, containing SV-40 large T antigen, driven by a BMP-2 promoter. When treated with osteogenic supplements, these cells mineralize and form bone matrix, expressing osteoblastic markers. The cells were plated at 1 x 10^6^ per T-150 flasks in Dulbecco`s Modified Eagle Medium (DMEM) supplemented with 10% heat-inactivated foetal bovine serum (HI-FBS; Lonza, UK), penicillin (100 U/ml), streptomycin (100 U/ml) and L-glutamine (Invitrogen, 2 mM) in a standard humidified incubator at 37°C and 5% CO_2_.

25(OH)D_2_, 1,25(OH)_2_D_2_, 25(OH)D_3_ and 1,25(OH)_2_D_3_ (Isosciences, CertiMass™) were dissolved in absolute ethanol at 10^−3^ M concentration as a stock solution, and stored in light-protected glass vials at -80°C. Working dilutions of 25(OH)D_2_ and 25(OH)D_3_ were evaluated by liquid chromatography-mass spectrometry. All sera used for tissue culture were routinely assessed for endogenous levels of 25(OH)D_2_ and 25(OH)D_3_.

To induce hBMSCs differentiation, these cells were plated in αMEM plus 10% HI-FBS to reach confluency, then supplemented with osteogenic medium (OSM, 10% HI-FBS, 10 mM β-glycerol 2-phosphate disodium salt and 50 μg/ml L-ascorbic acid (Sigma Aldrich, UK) and the addition of 10 nM dexamethasone. To promote differentiation of mouse 2T3 cells at confluency, the medium was changed to OSM. Cells were cultured in replicates in OSM in the presence of absence of various concentrations of 25(OH)D_2_/D_3_ (50–1000 nM) or 1,25(OH)_2_D_2_/D_3_ (0.05–10 nM) in ethanol as vehicle (total added volume was <1% of the culture medium volume). OSM media containing the vit D metabolites were changed every other day prior to RNA and protein extraction or until mineralisation was assessed.

### Cell number

Mouse 2T3 and hBMSCs were seeded in multiple 96-well plates at 5x10^3^ cell density per well (DMEM-10% with HI-FBS, and αMEM plus 10% HI-FBS, respectively) in replicates in the presence or absence of different concentrations of 25(OH)D_2_/D_3_ (50–1000 nM) or 1,25(OH)_2_D_2_/D_3_ (0.05–1 nM) for 24 h. After 21h, 10% MTS tetrazolium CellTiter^®^ One Solution Reagent (Promega) was added to the wells and incubated for the last 3h under standard tissue culture conditions. Plates were removed and growth was assayed at 490 nm using a SPECTRAmax plate reader (Molecular DEVICES, USA).

### ALP activity

Human BMSCs (1-5x10^4^) and mouse 2T3 cells (1x10^4^) were re-plated in replicates into 24/48 well plates in αMEM-10% HI-FBS and DMEM plus 10% HI-FBS culture media, respectively, to reach confluence, after which differentiation was induced by OSM in the presence or absence of vit D metabolites. For hBMSCs, the culture was for 7 days, during which the cells were treated every other day with either 100–1000 nM 25(OH)D_2_/D_3_ or 0.1–10 nM 1,25(OH)_2_D_2_/D_3_ in OSM. For 2T3 osteoblasts, the cells were cultured for 24 h, during which they were treated once with 100–500 nM 25(OH)D_2_/D_3_ or 0.1–0.5 nM 1,25(OH)_2_D_2_/D_3_ in OSM. Upon the completion of the treatment periods, cells were washed with Dulbeccos’s phosphate-buffered saline (DPBS) and lysed in 100 μl radioimmunoprecipitation assay buffer containing protease inhibitor (RIPA-PI; Sigma). Cell lysates were sonicated for 15 sec and total protein content was measured by bicinchoninic acid (BCA) protein assay kit (Thermo Scientific, UK). For each treated condition, ALP activities (pmol substrate/μg protein) of cell lysates were determined fluorometrically using fluorescent substrate 4-methylumbelliferyl phosphate (4-MUP; Sigma) as previously described [35]. Briefly, 10 μl of each cell lysate was incubated with 100 μl of freshly made 4MUP (0.16 mM in 50 mM Tris pH 6.0) substrate in white 96-well plates (NUNC) for 45 min in the dark at 37°C, followed by the addition of 100 μl of 0.6M Na_2_CO_3_ to stop the reaction. Fluorescence was measured at 360 nm excitation, 450 nm emission and 435 nm cut-off wavelength using a BMG Optima FluoSTAR plate reader (BMG LABTECH, Germany). ALP activities were calculated from standard curves of 0–20,000 pmol 4-methylumbelliferone (4MU), and values were normalised against the total protein content of each relevant well or treatment.

### Western blots

In order to determine the effects of the various vit D metabolites on endogenous levels of VDR, CYP24A1 and CYP27B1, as well as on the expression of osteoblast differentiation markers, OPN and OCN, hBMSCs were treated over 21 days with 1000 nM 25(OH)D_2_/D_3_ or 10 nM 1,25(OH)_2_D_2_/D_3_, and mouse 2T3 osteoblasts were treated for 8 days with either 100–500 nM 25(OH)D_2_/D_3_ or 0.01–0.5 nM 1,25(OH)_2_D_2_/D_3_ in OSM. Subsequently, cell lysates were extracted on ice in RIPA-PI. Total protein content under different treatment conditions was determined by BCA protein assay kit. Proteins (20 μg) were fractionated by 10–15% SDS-PAGE, transferred onto polyvinylidene difluoride (PVDF) membrane and incubated overnight separately with goat polyclonal anti-mouse/human OPN (R&D, UK; AF808 & AF1433), goat polyclonal anti-human OCN (Santa Cruz, USA; V19; sc-18319), goat polyclonal anti-mouse OCN (Santa Cruz, USA; G-20; sc-23790), mouse monoclonal anti-human/mouse VDR (Santa Cruz, USA; D-6; sc-13133), rabbit polyclonal anti-human CYP24A1 (Santa Cruz, USA; H-87; sc-66851), and rabbit polyclonal anti-mouse/human CYP27B1 (Santa Cruz, USA; H-90; sc-67261) primary antibodies (1:2000 to 1:4000 dilution). After washing, membranes were incubated for one hour with horseradish peroxidase-conjugated anti-goat IgG (R&D, UK; HAF017), anti-mouse (R&D, UK; HAF007) and anti-rabbit (Cell Signaling Technology, USA, #7074S) secondary antibodies (1:2000 diltuion), and immunoblots were visualised by enhanced chemiluminescence (ECL, Amersham, UK).

### RNA extraction, complementary DNA synthesis and RT-PCR

Human BMSCs were cultured at 1x10^5^ in αMEM-10% HI-FBS in replicates to reach confluence, when differentiation was induced by OSM in the presence or absence of 1000 nM concentrations of 25(OH)D_2_/D_3_ over 14 days. Mouse 2T3 cells were cultured in DMEM supplemented with 10% HI-FBS in 4-well plates at 1x10^5^ cell density until full confluence, followed by the addition of OSM in the presence or absence of 200 nM 25(OH)D_2_/D_3_. Total RNA from each treatment was extracted by RNeasy Mini Kit (Qiagen, UK) according to the manufacturer`s instructions. Nucleic acid concentrations were measured by NanoDrop ND-1000 spectrophotometer (Thermo Scientific, UK) at 260 nm. The absorbance ratios at 260/280 and 260/230 were used to detect any protein or organic carryover. Samples with both 260/280 and 260/230 ratios of ≥2 were used for further analysis. The integrity of total RNA purified with Qiagen RNeasy kit was also assessed using 1.5% agarose gel electrophoresis and ethidium bromide staining.

A total of 2 μg from each extract was treated with RNase-free DNase I (Thermo Scientific, UK) for 30 min at 37°C. Removal of genomic DNA was terminated by further heat inactivation with 100mM EDTA at 65°C for 10 mins. First strand cDNA synthesis of template RNA extracts was performed using a Veriti 96 well Thermal Cycler (Applied Biosystems, UK) using BIO-RAD iScript Reverse Transcription Supermix in a final reaction volume of 40 μl according to the manufacturer’s instruction.

RT-PCR was performed using a ViiA7 system (Applied Biosytems, UK) with commercially available lyophilized Quanititect Qiagen primers; *col1*, *opn*, *ocn*, *opg*, *rankl*, *vdr*, *cyp27b1*, *cyp24a1*. One μl of one-twentieth dilutions of templates were used in a total volume of 10 μl reaction in 384 well plates (Applied Biosystems, UK) by 2-step cycling (polymerase activation at 95°C for 2 min, 40 cycles of template denaturation at 95°C for 5 sec, primer annealing and extension at 65°C for 30 sec) using SYBR green Master Mix (BIOLINE; SensiFAST SYBR Lo-Rox Kit). The C_t_ values for treated samples were normalised to housekeeping genes *gapdh* and *18S* and the relative expressions were calculated using ^ΔΔ^C_t_ with amplification and accuracy of 98–100%.

### Mineralisation

Human BMSCs were cultured at 1x10^4^ in 96-well plates in αMEM-10% HI-FBS, and mouse 2T3 cells at 0.1-1x10^4^ in 96-, 48- or 24-well plates in replicates in DMEM-10%HI-FBS to reach confluency. Media at this time point (day zero) was replaced with OSM with or without vit D metabolites. For hBMSCs, OSM containing 500–1000 nM 25(OH)D_2_/D_3_ or 1–10 nM 1,25(OH)_2_D_2_/D_3_ was used, and for 2T3 cells, OSM contained 100–500 nM 25(OH)D_2_/D_3_ or 01–0.5 nM 1,25(OH)_2_D_2_/D_3_. The media were changed every other day up to 21 days for hBMSCs and up to 8 days for 2T3 cells prior to mineralisation assays. Upon completion of treatments, cell cultures were stopped, media removed, cells were washed with DPBS, fixed with 70% ethanol, air-dried and stained with 1.5% alizarin red dye solution (Sigma-Aldrich, UK; pH 4.1). Plates were washed with 70% ethanol and deionised water, air dried and staining were extracted by 10% acetic acid over orbit shaker, neutralised with 10% ammonium hydroxide, transferred into 96-well plates and measured colourimetrically at 550 nm.

### Statistical analysis

All experiments were carried out at least in triplicates and the mean ± SEM was calculated. Statistical analyses were carried out using SPSS version 11.0 for windows (SPSS Inc., Chicago, IL, USA). Effects of treatments were compared by Kruskal-Wallis one-way analysis of variance (ANOVA), with differences between treatments assessed using Bonferroni error protection for multiple comparisons. A *P* value of <0.05 was regarded to indicate a significant difference.

## Results

### Human BMS and mouse cell numbers

**Fig 1:**
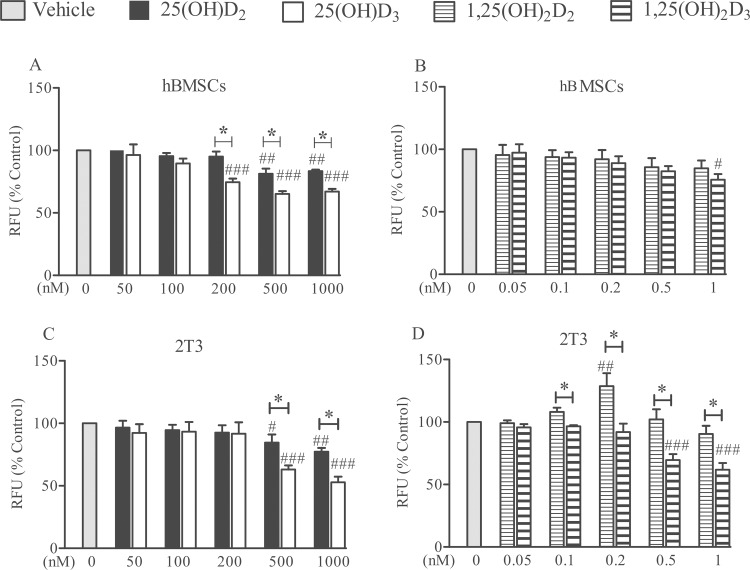
Comparative effects of 25(OH)D_2_, 25(OH)D_3_, 1,25(OH)_2_D_2_ and 1,25(OH)_2_D_3_ on cell numbers. Human BMSCs (panels A&B) and 2T3 cells (panels C&D) received separate treatments over 24 h with either 50–1000 nM 25(OH)D_2_/D_3_ or 0.05–1 nM 1,25(OH)_2_D_2_/D_3_. Cell numbers were measured by MTS. Mean ± SEM percentage values from triplicate experiments have been presented as fluorescence units (RFU) from treated cells relative to untreated control (vehicle). # *P*<0.05, ## *P*<0.01, ### *P*<0.001 for comparisons between the treatments and the control; * *P*<0.01 for comparisons between the D_2_ and D_3_ metabolites.

Human BMSC numbers decreased in response to 25(OH)D_2_ at doses of ≥500 nM (and in response to 25(OH)D_3_ at doses of ≥200 nM (*P*<0.01 and *P*<0.001, respectively; Fig 1A). 25(OH)D_3_ was more potent than D_2_ at concentrations of ≥200 nM in human primary cells (*P*<0.01; Fig 1A). Only a small decrease in hBMSC numbers as a result of treatment with the highest dose of 1,25(OH)_2_D_3_ (1 nM) was observed (*P*<0.05; Fig 1B).

When mouse 2T3 osteoblasts were exposed to 25(OH)D_2_, cell numbers were reduced at doses of 500 nM and 1000 nM (*P*<0.05 and *P*<0.01, respectively; Fig 1C). A decrease in 2T3 cell numbers was also observed with 25(OH)D_3_ at doses of 500 nM and 1000 nM (*P*<0.001 for both concentrations; Fig 1C). The magnitude of the decrease was greater with 25(OH)D_3_ compared with 25(OH)D_2_ (*P*<0.01; Fig 1C). In 2T3 cells, whereas 1,25(OH)_2_D_3_ at ≥0.5 nM concentrations reduced cell numbers significantly (*P*<0.001; Fig 1D), 1,25(OH)_2_D_2_ had no such effects, and, if anything, showed a tendency to increase cell numbers only at 0.2 nM (*P*<0.01; Fig 1D).

### ALP in hBMSCs and mouse osteoblasts

**Fig 2:**
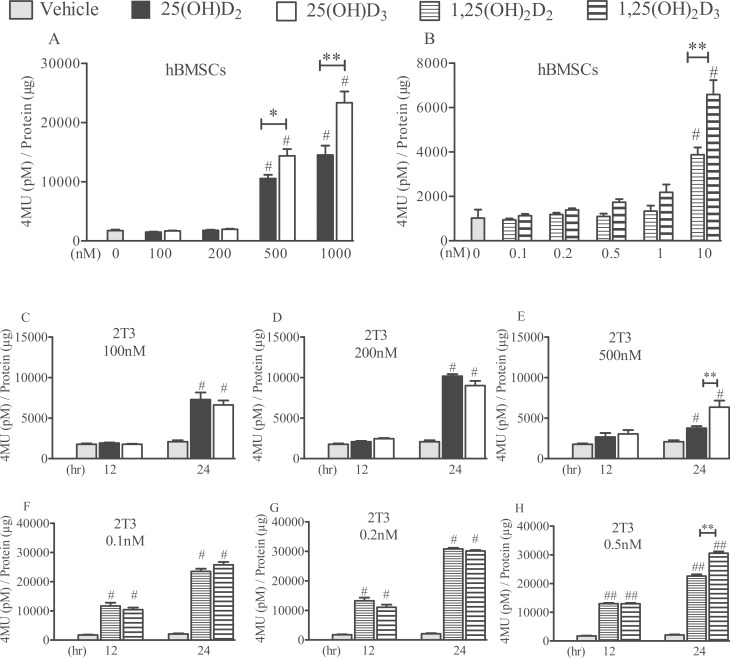
Comparative effects of 25(OH)D_2_, 25(OH)D_3_, 1,25(OH)_2_D_2_ and 1,25(OH)_2_D_3_ on ALP enzyme activity. Human BMSCs were grown in αMEM-10% FBS for 4 days until confluency (day 0) and treated with the D_2_ or D_3_ metabolites every other day (days 0, 2, 4 and 6). Human BMSC cultures were stopped at day 7, and ALP activity was determined (panels A&B). 2T3 cells were grown in 10% DMEM-FBS for 24 h until full confluency (day 0), and were treated once with either 100–500 nM 25(OH)D_2_/D_3_ (panels C-E) or 0.1–0.5 nM 1,25(OH)_2_D_2_/D_3_ (panels F&H) in differentiation media. Cultures were stopped at 12 or 24 h, and ALP activity was measured. ALP activities were normalised to total protein content. Mean ± SEM enzymatic activity values from triplicate experiments have been presented as the amount of 4-methylumbelliferone (4MU) generated after 45 min at 37°C. # *P*<0.001 for comparisons between the treatments and the control (vehicle); * *P*<0.05 and ** *P*<0.01 for comparisons between the D_2_ and D_3_ metabolites.

In hBMSCs, ALP activity increased after 7 days of treatment with 500–1000 nM 25(OH)D_2_ and 25(OH)D_3_ compared with controls (*P*<0.001; Fig 2A). After a similar period of treatment with 1,25(OH)_2_D_2_ and 1,25(OH)_2_D_3_ but only at a dose of 10 nM, ALP activity also increased compared with controls (*P*<0.001; Fig 2B). Human BMSCs displayed a differential response to D_2_ versus D_3_ metabolites, with greater potencies observed in the case of 25(OH)D_3_ (*P*<0.05 at 500 nM and *P*<0.01 at 1000 nM; Fig 2A) and 1,25(OH)_2_D_3_ (*P*<0.01 for 1 nM; Fig 2B).

At 100–500 nM concentrations, both 25(OH)D_2_ and 25(OH)D_3_ also increased ALP activity in 2T3 cells after 24 h of treatment (*P*<0.001; Fig 2C–2E), with 25(OH)D_3_ being more potent than 25(OH)D_2_ at 500 nM concentration (*P*<0.01; Fig 2E). In the same cells, 0.1–0.5 nM 1,25(OH)_2_D_2_ and 1,25(OH)_2_D_3_ also induced an increase in ALP compared with controls (*P*<0.001; Fig 2F–2H). At 24 h and at a concentration of 0.5 nM, 1,25(OH)_2_D_3_ also displayed a greater potency than 1,25(OH)_2_D_2_ (*P*<0.01; Fig 2H) in mouse 2T3 cells.

### Relative effects of 25(OH)D_2_ and 25(OH)D_3_ on gene expression

**Fig 3:**
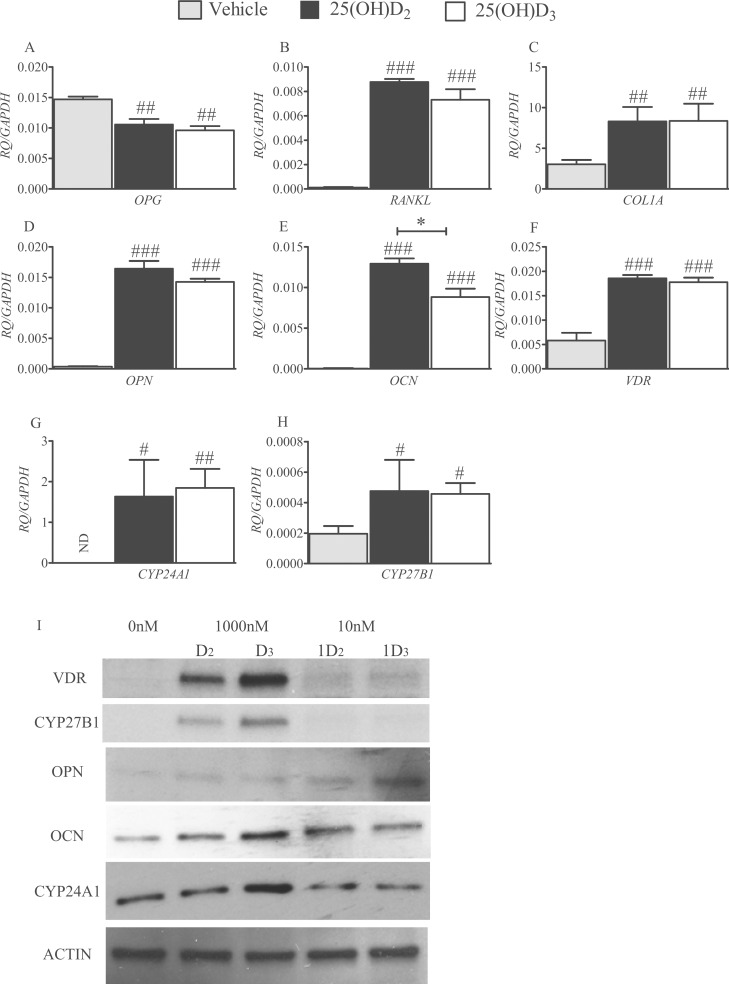
Effects of 25(OH)D_2_ and 25(OH)D_3_ on hBMSC gene expression. Histograms depict comparative effects of 25(OH)D_2_ and 25(OH)D_3_ on the expression of hBMSC signature genes, *opg* (A), *rankl* (B), *col1a* (C), *opn* (D), *ocn* (E), *vdr* (F), *cyp24a1* (G) *and cyp27b1* (H), during their differentiation. Human BMSCs were cultured for 14 days in the presence or absence of 1000 nM of either 25(OH)D_2_ or 25(OH)D_3_ in differentiation media. Quantified values (RQ) have been normalised to *gapdh* expression and are mean ± SEM from triplicate experiments. # *P*<0.05, ## *P*<0.01 and ### *P*<0.001 for comparisons between 25(OH)D_2_ or 25(OH)D_3_ treatments and the control (vehicle); * *P*<0.01 for comparisons between 25(OH)D_2_ and 25(OH)D_3_. ND = not detectable. Western blots showing relative amounts of VDR, CYP27B1, OPN, OCN and CYP24A1 proteins in hBMSC cell lysates (20 μg protein) after 21 days of treatment with 1000 nM of either 25(OH)D_2_ (D_2_) or 25(OH)D_3_ (D_3_), or 10 nM of either 1,25(OH)_2_D_2_ (1D_2_) or 1,25(OH)_2_D_3_ (1D_3_) (I).

Treatments of hBMSCs with 1000 nM of 25(OH)D_2_ and 25(OH)_2_D_3_ suppressed *opg* expression equally (*P*<0.01; Fig 3A), and had comparable effects in up-regulating transcriptions of *rankl* (*P*<0.0001; Fig 3B), *col1a* (*P*<0.01; Fig 3C), *opn* (*P*<0.001; Fig 3D), *vdr* (*P*<0.001; Fig 3F), *cyp24a1* (*P*<0.05 for D_2_ and *P*<0.01 for D_3_; Fig 3G) and *cyp27b1* (*P*<0.05; Fig 3H) compared with controls. 25(OH)D_2_ was more potent than 25(OH)D_3_ in up-regulation of *ocn* in hBMSCs (*P*<0.01; Fig 3E).

**Fig 4:**
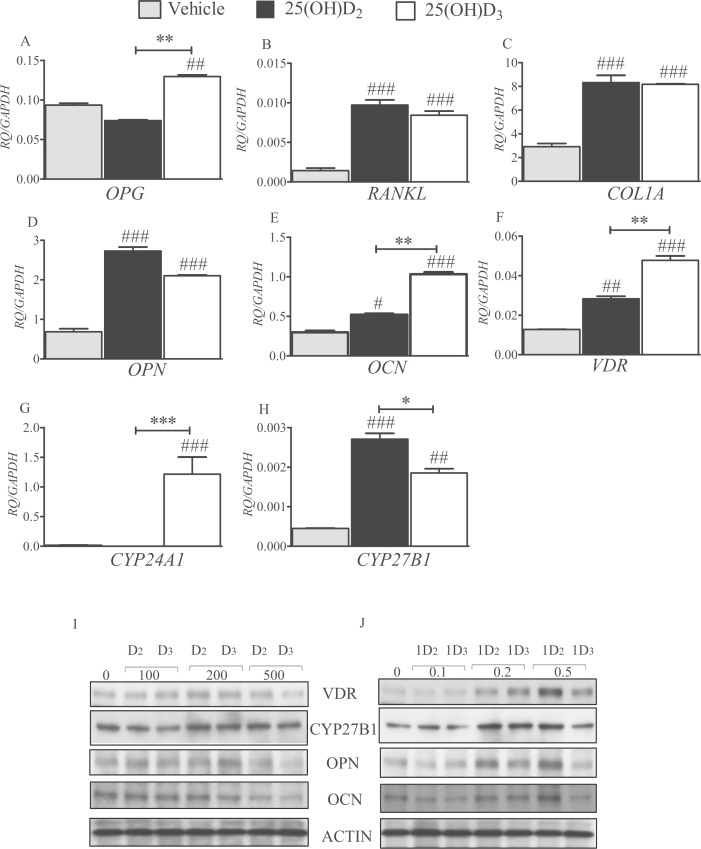
Effects of 25(OH)D_2_ and 25(OH)D_3_ on 2T3 cell gene expression. Histograms depict the comparative effects of 25(OH)D_2_ and 25(OH)D_3_ on the expression of mouse 2T3 osteoblast signature genes, *opg* (A), *rankl* (B), *col1a* (C), *opn* (D), *ocn* (E), *vdr* (F), *cyp24a1* (G) *and cyp27b1* (H). 2T3 cells were cultured for 24 h in the presence or absence of 200 nM of either 25(OH)D_2_ or 25(OH)D_3_ in differentiation media. Quantified values (RQ) have been normalised to *gapdh* expression and are mean ± SEM from triplicate experiments. # *P*<0.05, ## *P*<0.01 and ### *P*<0.001 for comparisons between 25(OH)D_2_ or 25(OH)D_3_ treatments and the control (vehicle); * *P*<0.05, ** *P*<0.01 and *** *P*<0.001 for comparisons between 25(OH)D_2_ and 25(OH)D_3_. Western blots showing relative amounts of VDR, CYP27B1, OPN and OCN proteins in 2T3 cell lysates (20 μg protein) after 8 days of treatment with (I) 100–500 nM of either 25(OH)D_2_ (D_2_) or 25(OH)D_3_ (D_3_), and (J) 0.1–0.5 nM of either 1,25(OH)_2_D_2_ (1D_2_) or 1,25(OH)_2_D_3_ (1D_3_).

When murine 2T3 osteoblasts were treated with 200 nM of 25(OH)D_2_ or 25(OH)D_3_ for 24 h in osteogenic media, there was an increase in the transcription of *opg* (*P*<0.01 for D_3_; Fig 4A), *rankl* (*P*<0.001 for both metabolites; Fig 4B), *col1a* (*P*<0.001 for both metabolites; Fig 4C), *opn* (*P*<0.001 for both metabolites; Fig 4D), *ocn* (*P*<0.05 for 25(OH)D_2_ and *P*<0.001 for 25(OH)D_3_; Fig 4E), *vdr* (*P*<0.01 for 25(OH)D_2_ and *P*<0.001 for 25(OH)D_3_; Fig 4F) and *cyp27b1* (*P*<0.001 for 25(OH)D_2_ and *P*<0.01 for 25(OH)D_3_; Fig 4H) compared with controls. There was no detectable change in *cyp24a1* expression in response to 25(OH)D_2_ over 24 h, whereas 25(OH)D_3_ significantly up-regulated *cyp24a1* transcription by around 70-fold (*P*<0.001; Fig 4E). For *opg*, *ocn* and *vdr*, 25(OH)D_3_ had a significantly greater influence than 25(OH)D_2_ on gene transcription (*P*<0.01 for all comparisons; Fig 4A, 4E and 4F). However, 25(OH)D_2_ was more potent than 25(OH)D_3_ in inducing *cyp27b1* transcription (*P*<0.05; Fig 4H).

### Western blot

In hBMSCs, the expression of VDR, CYP27B1, OCN and CYP24A1 proteins appeared to be up-regulated to a greater extent in response to the addition of 1000 nM 25(OH)D_3_ than 25(OH)D_2_ (Fig 3I). When hBMSCs were treated with 10 nM 1,25(OH)_2_D_2_ or 1,25(OH)_2_D_3,_ both metabolites up-regulated VDR, OCN and CYP24A1 equally (Fig 3I). Neither metabolite had any effects on CYP24A1. 1,25(OH)_2_D_3_ appeared more potent that 1,25(OH)_2_D_2_ in up-regulating OPN protein in hBMSCs (Fig 3I).

In 2T3 cells treated with 25(OH)D_2_ or 25(OH)D_3_, there were moderate increases in VDR, CYP27B1, OPN and OCN at 200 nM (Fig 4I). However, a reduction of these proteins was observed with 500 nM of both 25(OH)D_2_ and 25(OH)D_3_, with the latter being more potent in this reduction (Fig 4I). There were dose-dependent increases in VDR, OPN and OCN protein expression in response to 1,25(OH)_2_D_2_ and 1,25(OH)_2_D_3_, where 0.2–0.5 nM 1,25(OH)_2_D_2_ elicited the greatest response (Fig 4J).

### Mineralisation

**Fig 5:**
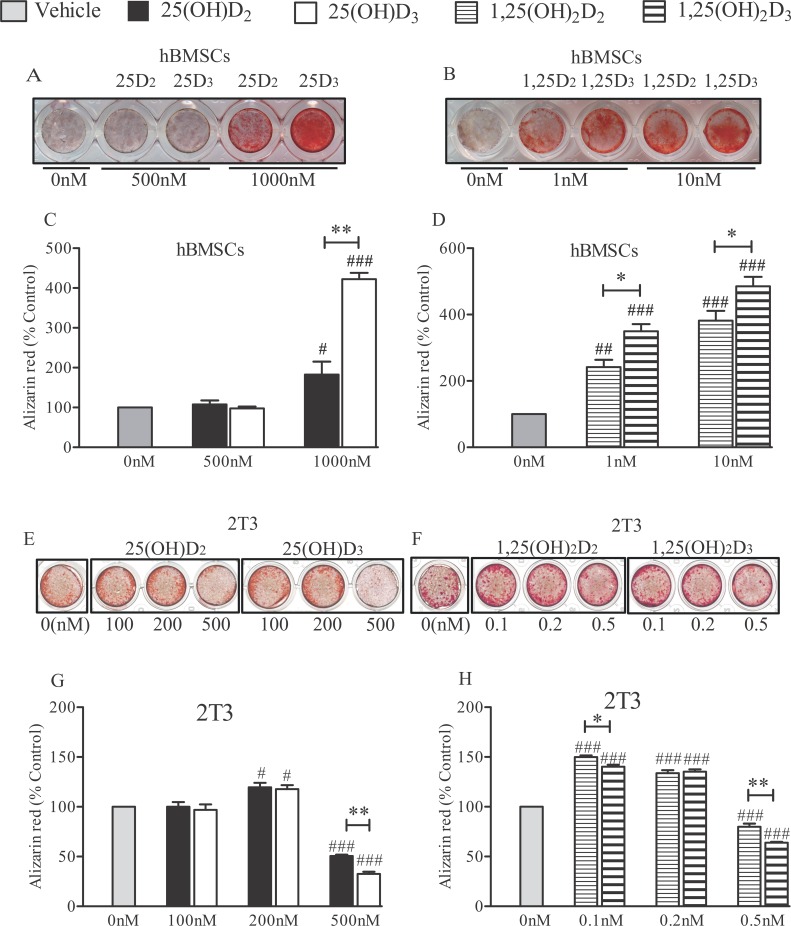
Effects of 25(OH)D_2_, 25(OH)D_3_, 1,25(OH)_2_D_2_ and 1,25(OH)_2_D_3_ on mineralisation. Human BMSCs cells were cultured in differentiation media for 21 days in the presence of either 500–1000 nM 25(OH)D_2_/D_3_ (A) or 1–10 nM 1,25(OH)_2_D_2_/D_3_ (B), stained with alizarin red and photographed. Quantification of mineralisation was by alizarin red stain extraction and colourimetry, and the effects of treatment of hBMSCs with either 500–1000 nM 25(OH)D_2_/D_3_ (C) or 0.1–10 nM 1,25(OH)_2_D_2_/D_3_ (D) are presented. Values are mean ± SEM from triplicate experiments as percentage of untreated controls (vehicle). 2T3 cells were cultured in differentiation media for 8 days in the presence of either 100–500 nM 25(OH)D_2_/D_3_ (E) or 0.1–0.5 nM 1,25(OH)_2_D_2_/D_3_ (F). Alizarin red staining of 2T3 was quantified and the responses to 100–500 nM 25(OH)D_2_/D_3_ (G) or 0.1–0.5 nM 1,25(OH)_2_D_2_/D_3_ (H) are shown as mean ± SEM from triplicate experiments and as percentage of untreated controls. # *P*<0.05, ## *P*<0.01 and ### *P*<0.001 for comparisons between the treatments and the control; * *P*<0.05 and ** P<0.001 for comparisons between the D_2_ and D_3_ metabolites.

When hBMSCs were cultured in osteogenic media for 21 days and treated with the vit D metabolites every other day, no mineralisation was observed at concentrations of 500 nM of 25(OH)D_2_ or 25(OH)D_3_ (Fig 5A). Only at a concentration of 1000 nM 25(OH)D_2_ and D_3_ was mineralisation significantly increased in these cells (*P*<0.05 and *P*<0.001, respectively), and the effects were more pronounced with the 25(OH)D_3_ (*P*<0.001; Fig 5A and 5C). Quantified alizarin red staining revealed that 1–10 nM 1,25(OH)_2_D_3_ was more potent in mineralizing hBMSCs than 1,25(OH)_2_D_2_ (*P*<0.05; Fig 5B and 5D).

Quantified alizarin red staining revealed that 200 nM 25(OH)D_2_ and 25(OH)D_3_ equally increased mineralisation in 2T3 (*P*<0.05; Fig 5G). There was also an increase in 2T3 mineralisation in response to both 1,25(OH)_2_D_2_ and 1,25(OH)_2_D_3_ at 0.1–0.2 nM (*P*<0.001), with 0.1 nM 1,25(OH)_2_D_2_ being more stimulatory than D_3_ (*P*<0.05; Fig 5H). 25(OH)D_2_ and 25(OH)D_3_ at doses of 500 nM, and also 1,25(OH)_2_D_2_ and 1,25(OH)_2_D_3_ at 0.5 nM significantly inhibited mineralisation in 2T3 osteoblasts *(P*<0.001; Fig 5E and 5F). This inhibitory effects was more evident with 25(OH)D_3_ and with 1,25(OH)_2_D_3_ than the corresponding D_2_ metabolites (*P*<0.001; Fig 5G and 5H).

## Discussion

To the best of our knowledge, this is the first study that has compared the effects of 25(OH) or 1,25(OH)_2_ metabolites of D_2_ and D_3_ on osteoblasts. Unlike previous studies that have employed pharmacologically high concentrations of 25(OH) and 1,25(OH)_2_ metabolites, we used concentrations that were generally much lower and more physiological [4,7,9,15]. Differences in the effects of these metabolites on osteoblastic cells from human and mouse that were also very distinct in their stage of differentiation were studied. Most previous studies have shown the effects of vit D metabolites on osteoblast proliferation to be inhibitory [14,36,37].

In hBMSCs, 25(OH)D_3_ had a greater inhibitory effect on cell number than 25(OH)D_2_, and with 1,25(OH)_2_ metabolites, an inhibition was seen only with 1,25(OH)_2_D_3_ at a concentration of 1000 pmol/L. We also found both 25(OH)D and 1,25(OH)_2_D metabolites to inhibit increasing 2T3 cell number, and the suppressive effects to be greater with the D_3_ metabolites. A recent study on human osteoblasts have shown 1,25(OH)_2_D_3_ metabolite to decrease cell proliferation, but only at a pharmacologically high concentration of 100 nmol/L administered over 3 days [15]. Our data show that over a period of 24 h, some stasis in cell number, albeit small, appear to be present at lower vit D metabolite concentrations, particularly with those of D_3_. Such anti-proliferative effects are achieved by cell cycle arrest, with the effects of 25(OH)D presumably mediated through the activity of CYP27B1 and the generation of 1,25(OH)_2_ [38].

During the differentiation of hBMSCs into osteoblasts, their VDR expression increases [39]. Our observations on VDR gene and protein expression lend support to this, particularly in relation to stimulation by 25(OH)D metabolites, with 25(OH)D_3_ having a greater effect on the VDR protein. Activated VDR acts as a transcription factor for osteoblast signature genes, and in hBMSCs, active vit D metabolites stimulate the manufacture of bone matrix proteins that include Col1, OCN and OPN, and increase ALP production that promotes mineralisation [7,13,40,41]. Our data in hBMSCs are consistent with such previous findings, with both 25(OH)D metabolites increasing the transcription of *col1*, *ocn* and *opn* matrix protein genes, as well as that of *opn*, *rankl*, and *cyp24a1* and *cyp27b1*. Our data showed a greater effect of 25(OH)D_3_ than 25(OH)D_2_ in increasing the manufacture of ALP, OCN, CYP24A1 and CY27B1 proteins.

It is unclear why 1,25(OH)_2_ metabolites did not affect hBMSC cell number or stimulate VDR, CYP24A1 and CYP27B1 as much as 25(OH) metabolites did. Whether this may be related to higher 1,25(OH)_2_D concentrations produced at cellular level from nM range of 25(OH)D used is unknown, although some previous findings seem to suggest this [38]. An increase in CYP27B1 increases 1,25(OH)_2_D, and CYP24A1 converts 25(OH)D and, in particular, 1,25(OH)_2_D metabolites into their 24-hydroxylated forms [42]. It has been shown that both 24,25(OH)_2_D and particularly 1,24,25(OH)_3_D are biologically active and exert stimulatory effects on vit D-responsive cells [43,44], but the exact influence of these metabolites on osteoblasts remains unknown.

Unlike the scenario in hBMSCs, in 2T3 cells 25(OH)D_3_ was a more potent stimulus to *vdr* and *ocn* gene expression than 25(OH)D_2_, but both VDR and OCN proteins were higher with 1,25(OH)_2_D_2_ compared with 1,25(OH)D_3_. As with previous reports [38], we also found 25(OH) metabolites to increase *cyp27B1* gene expression in 2T3 cells, but in contrast to human cells, this was greater with 25(OH)D_2_. In contrast to hBMSCs, 2T3 cells produced large amounts of CYP27B1 enzyme in response to 1,25(OH)_2_D_2_. Furthermore, unlike hBMSCs, mouse cells expressed *cyp24A1* gene only in response to 25(OH)D_3_ and not 25(OH)D_2_. Whereas the influence of CYP27B1 expression is consistent with the cell number and mineralisation effects of vit D as described above, the physiological effects of stimulation of CYP24A1 is uncertain.

Both 25(OH)D_2_ and 25(OH)D_3_ greatly increased *rankl* gene transcription in human and mouse cells. The two metabolites modestly suppressed *opg* gene transcription in hBMSCs, but had inconsistent effects in 2T3 cells. RANKL and OPG exert opposing effects on osteoclastogenesis and osteoclast activation, and a pattern of protein expression similar to that of gene expression observed in human cells here would be a stimulus to osteoblast activation [45–47]. With regards mineralisation as a crucial clinical endpoint of vit D effect in relation to bone mineral density, hBMSC’s mineralisation increased with vit D metabolites. This was in accord with our ALP data, and these effects tended to be greater with 25(OH)D_3_ and 1,25(OH)D_3_ at high concentrations. These add further support to a prevailing general notion that D_2_ treatment has less effect than D_3_ administration in man.

However, previous data in some murine osteoblasts have shown that, unlike its effects on human cells, vit D metabolites appear to inhibit differentiation and mineralisation, although studies on murine ALP and Col1 show some conflicting data [9,48–51]. Our observations on 2T3 mineralisation indicate a bimodal concentration effect of vit D metabolites in these cells. Thus, there was an increase in mineralisation at low vit D metabolites concentrations, but the effects were reversed and became suppressive as concentrations increased. These data may suggest that some of the inhibitory effects on mineralisation observed in some previous studies may have been due to the use of very high, supra-physiological doses of these metabolites in those studies. At high concentrations, the suppressive effects of vit D metabolites on 2T3 cell mineralisation was greater with 25(OH)D_3_ and 1,25(OH)_2_D_3_ than the corresponding D_2_ compounds. Two further murine cell lines, MBA 15.4 and Mc3T3 E1, displayed similar dose responses (data not shown), with enhanced differentiation over doses 100–200 nM and repression at ≥500 nM.

These findings indicate that cell activity can change significantly over time under the influence of active vit D metabolites. In that respect, two different concentrations of a vit D metabolite may be able to bring about two patterns of change in cell activity that are out of synch with one another during their time course. For this reason, it is possible that some of the conflicting differences in the expression of a particular protein seen with D_3_ and D_2_ metabolites may be secondary to a greater potency of one metabolite that imposes a shift in the time course of change in that protein. The data on such a protein can then become conflicting when there is both an increase and a decrease in protein expression over a time course. Such apparent conflicts may be possible in our study as we assessed gene expression over long periods of 1 and 14 days of treatment of 2T3 and hBMS cells, respectively. Furthermore, the effects of vit D metabolites on osteoblasts are known to be influenced by other factors such as calcium concentrations [52]. How these other factors change over time is unknown, but they can potentially add further complexity to the dynamics of the effects of vit D metabolites that may possess different potencies.

Overall, the data from this study suggest that in general vit D_3_ metabolites appear to elicit a greater influence on both hBMSC and mouse 2T3 cells. These greater effects appear not to be universal, but seem to be more pronounced in some major aspects of osteoblast function, particularly in changing cell number and in increasing ALP and mineralisation. The only greater influences of 25(OH)D_2_ that we found were on OCN expression in hBMSCs and on CYP27B1 expression in 2T3 calls. Assuming that the effects of 25(OH)D are mostly mediated through the generation of its α-hydroxylated compound by CYP27B1, as well as the metabolism of 25(OH)D and 1,25(OH)_2_D by CYP24A1, consideration must be given to the possibility that the velocity and the Km of these enzymes may differ significantly for 25(OH)D_2_ and 25(OH)D_3_.

Furthermore, some of the differences in the effects of vit D_3_ metabolites on osteoblast proliferation and function appear to be also time- and cell-type-dependent [10]. The same may be true for the effects of D_2_ metabolites. As with others, we have observed that the direction and the effects of vit D metabolites can differ sufficiently in the two osteoblastic cell types studied so as to preclude the use of mouse model to derive knowledge about human cells. In some respects, the data on mouse osteoblasts may in fact be even misleading in predicting the behaviour of human cells. However, human osteosarcoma cell lines display similarly diverse responses, with neither MG63 nor SaOS2 cell lines responding to the same dose and range of vitamin D as primary hBMSCs with either ALP activity or mineralisation (S1 Fig in supporting information).

In conclusion, our data on osteoblasts add significant weight to some previous findings that D_2_ may not have the same potency and may not elicit the same physiological effect as D_3_. If corroborated by further future studies, these findings would underline a need to assess differences in long-term physiological effects of the two vitamins in human subjects. Evidence gathered from such studies may prove to have implications for prescribing D_2_ preparations in clinical practice.

## Supporting Information

S1 File Raw data for Figs 1–5. The raw data, based on which Figs 1–5 have been drawn, have been presented in Tabs 1–5 of the file, respectively.
(XLS)

S1 Fig Effects of 25(OH) and 1,25(OH)_2_ metabolites of D_2_ and D_3_ on ALP enzyme activity (page 1) and mineralisation (page 2) of MG-63 and SaOS-2 cells. (PPT)
