# Supplementary material for: 25-Hydroxy- and 1α,25-Dihydroxycholecalciferol Have Greater Potencies than 25-Hydroxy- and 1α,25-Dihydroxyergocalciferol in Modulating Cultured Human and Mouse Osteoblast Activities
Source: PLoS One. 2016 Nov 28;11(11):e0165462. doi: 10.1371/journal.pone.0165462 (PMC5125576; doi:10.1371/journal.pone.0165462)
Supplement: S1 Fig — (PPT) [file pone.0165462.s002.ppt]

## Slide 1
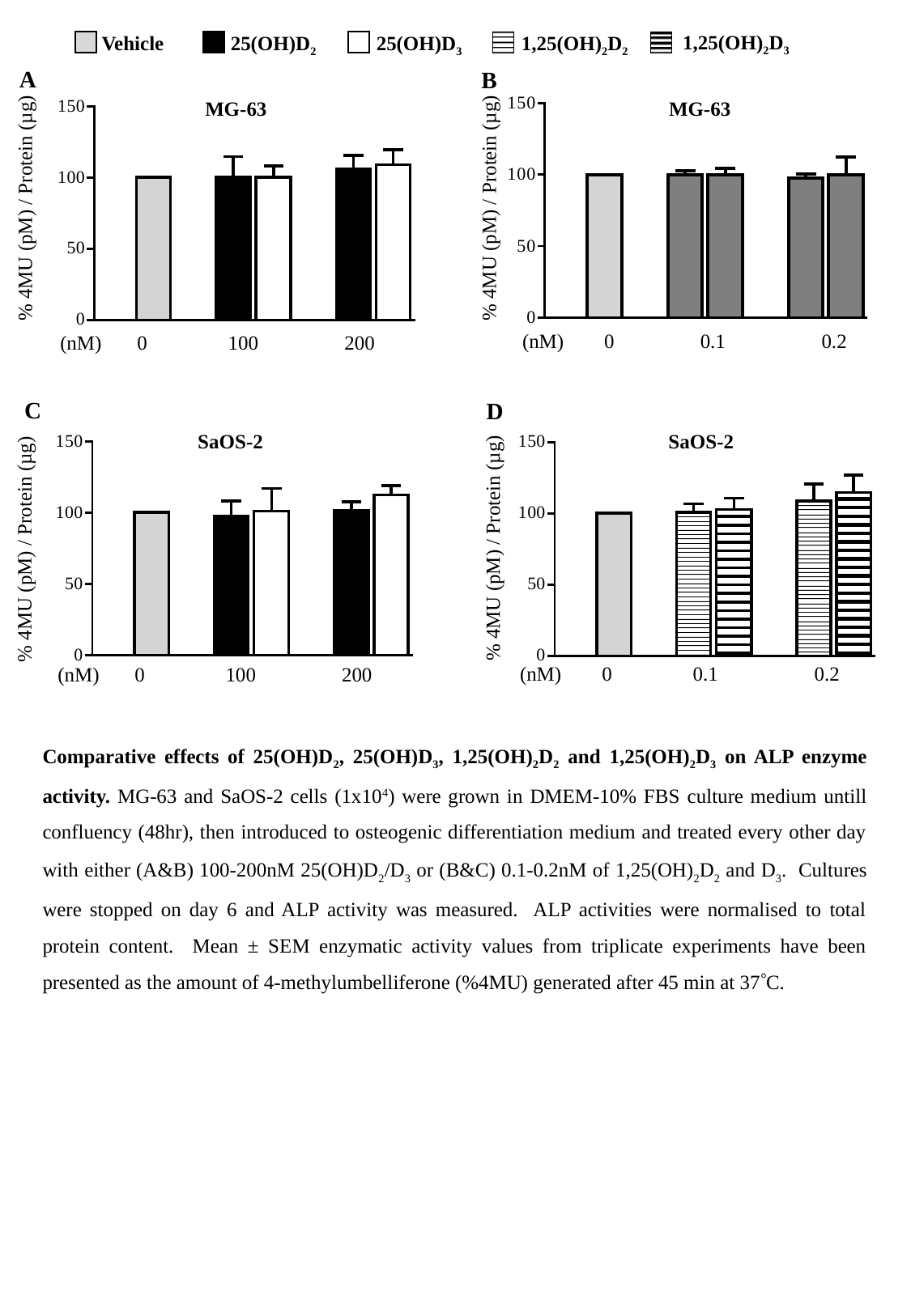

1,25(OH)2D3
Vehicle
25(OH)D2
25(OH)D3
1,25(OH)2D2
A
B
MG-63
MG-63
% 4MU (pM) / Protein (µg)
% 4MU (pM) / Protein (µg)
 (nM) 0 100 200
(nM) 0 0.1 0.2
C
D
SaOS-2
SaOS-2
% 4MU (pM) / Protein (µg)
% 4MU (pM) / Protein (µg)
 (nM) 0 100 200
(nM) 0 0.1 0.2
Comparative effects of 25(OH)D2, 25(OH)D3, 1,25(OH)2D2 and 1,25(OH)2D3 on ALP enzyme activity. MG-63 and SaOS-2 cells (1x10^4^) were grown in DMEM-10% FBS culture medium untill confluency (48hr), then introduced to osteogenic differentiation medium and treated every other day with either (A&B) 100-200nM 25(OH)D2/D3 or (B&C) 0.1-0.2nM of 1,25(OH)2D2 and D3. Cultures were stopped on day 6 and ALP activity was measured. ALP activities were normalised to total protein content. Mean ± SEM enzymatic activity values from triplicate experiments have been presented as the amount of 4-methylumbelliferone (%4MU) generated after 45 min at 37°C.

## Slide 2
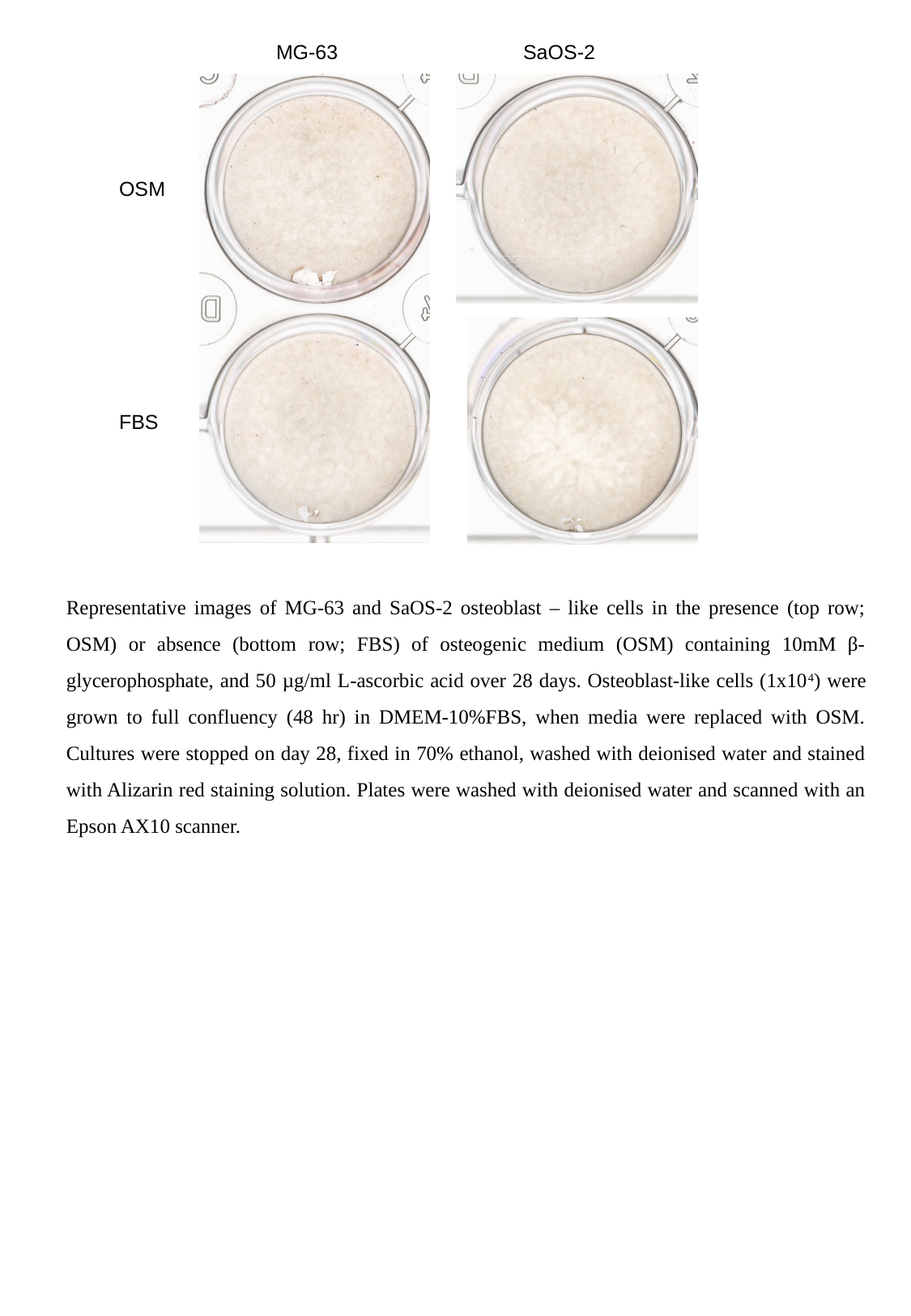

MG-63
SaOS-2
OSM
FBS
Representative images of MG-63 and SaOS-2 osteoblast – like cells in the presence (top row; OSM) or absence (bottom row; FBS) of osteogenic medium (OSM) containing 10mM β-glycerophosphate, and 50 µg/ml L-ascorbic acid over 28 days. Osteoblast-like cells (1x10^4^) were grown to full confluency (48 hr) in DMEM-10%FBS, when media were replaced with OSM. Cultures were stopped on day 28, fixed in 70% ethanol, washed with deionised water and stained with Alizarin red staining solution. Plates were washed with deionised water and scanned with an Epson AX10 scanner.
